# Training recruiters to randomized trials to facilitate recruitment and informed consent by exploring patients' treatment preferences

**DOI:** 10.1186/1745-6215-15-323

**Published:** 2014-08-13

**Authors:** Nicola Mills, Jane M Blazeby, Freddie C Hamdy, David E Neal, Bruce Campbell, Caroline Wilson, Sangeetha Paramasivan, Jenny L Donovan

**Affiliations:** School of Social and Community Medicine, University of Bristol, Canynge Hall, Bristol, 39 Whatley Road, BS8 2PS UK; Nuffield Department of Surgery, University of Oxford, John Radcliffe Hospital, Headley Way, Headington, Oxford, OX3 9DU UK; University Department of Oncology, Addenbrooke’s Hospital, Hills Road, Cambridge, CB2 0QQ UK; Royal Devon and Exeter Hospital, University of Exeter Medical School, Barrack Road, Exeter, EX2 5DW UK

**Keywords:** Treatment preferences, Qualitative research methods, Randomized controlled trials, Recruitment to randomized controlled trials, Informed consent, ProtecT study

## Abstract

**Background:**

Patients’ treatment preferences are often cited as barriers to recruitment in randomized controlled trials (RCTs). We investigated how RCT recruiters reacted to patients’ treatment preferences and identified key strategies to improve informed decision-making and trial recruitment.

**Methods:**

Audio-recordings of 103 RCT recruitment appointments with 96 participants in three UK multicenter pragmatic RCTs were analyzed using content and thematic analysis. Recruiters’ responses to expressed treatment preferences were assessed in one RCT (ProtecT - Prostate testing for cancer and Treatment) in which training on exploring preferences had been given, and compared with two other RCTs where this specific training had not been given.

**Results:**

Recruiters elicited treatment preferences similarly in all RCTs but responses to expressed preferences differed substantially. In the ProtecT RCT, patients’ preferences were not accepted at face value but were explored and discussed at length in three key ways: eliciting and acknowledging the preference rationale, balancing treatment views, and emphasizing the need to keep an open mind and consider all treatments. By exploring preferences, recruiters enabled participants to become clearer about whether their views were robust enough to be sustained or were sufficiently weak that participation in the RCT became possible. Conversely, in the other RCTs, treatment preferences were often readily accepted without further discussion or understanding the reasoning behind them, suggesting that patients were not given the opportunity to fully consider all treatments and trial participation.

**Conclusions:**

Recruiters can be trained to elicit and address patients’ treatment preferences, enabling those who may not have considered trial participation to do so. Without specific guidance, some RCT recruiters are likely to accept initial preferences at face value, missing opportunities to promote more informed decision-making. Training interventions for recruiters that incorporate key strategies to manage treatment preferences, as in the ProtecT study, are required to facilitate recruitment and informed consent.

**Trial registration:**

ProtecT RCT: Current Controlled Trials ISRCTN20141297. The other two trials are registered but have asked to be anonymized.

## Background

Recruitment to randomized controlled trials (RCTs) is often challenging, with less than 50% of trials estimated to meet recruitment targets [1]. Patients’ treatment preferences have been frequently cited as a barrier to trial recruitment [2–5] and clinicians find it difficult to recruit patients who favor a particular treatment [6–9]. Several studies have focused on the impact of patients’ treatment preferences on trial recruitment and outcome [4, 10], but there has been little attention to what constitutes a preference and how it should be measured and handled within a trial setting or in clinical practice.

Several previous studies have tended to assume that patients’ treatment preferences are simple and static entities that can be easily defined and measured [4, 10]. However, there is also a small body of research that shows that preferences are complex, multifaceted, and changeable entities that can be based on incomplete or inaccurate information [11–16]. Within the context of RCTs, a treatment preference has been defined as ‘the difference in the perceived desirability of two or more interventions’ - a relative and potentially quantifiable concept by which the desirability of one intervention is compared with another [16]. Within this definition, and within the wider literature on ‘desirability’, preferences can be broadly based on expectancies concerning the process and outcomes associated with the intervention and the perceived value placed on those outcomes and processes [16]. Various factors can shape preferences including the way in which the information on interventions is presented or ‘framed’ [17–21], prior experience of treatment [13], and whether or not the treatment is available outside of the trial [22]. Patient sociodemographic and health status can also determine treatment preferences, for example younger patients have been shown to prefer a more aggressive approach to treatment than older patients [23, 24], as do those who have ‘someone to live for’ [25]. The study of preferences is further complicated by their instability; preferences may not be fixed and can change as experience of an intervention is gained [16]. In our previous research into the role of patients’ treatment preferences at trial recruitment, we exposed the difficulty of defining and studying treatment preference due to the variation in the way it was expressed between participants and over time, and how it changed in response to interactions with recruiters [15].

Eliciting patients’ treatment preferences has been proposed as one of the key elements to ensure shared decision-making in clinical practice [26]. Stiggelbout et al. state that clinicians should determine patients’ treatment preferences ‘to judge whether the benefits and risks of treatments are balanced from the patient’s perspective and to avoid procedures patients would rather not have if they are well-informed’ [27]. This approach is also important in RCT recruitment, where the aim is to give potential recruits sufficient information about the treatment options within and outside of the trial to make an informed decision about participation or otherwise [28]. Our previous research in an RCT for localised prostate cancer treatment (the ProtecT - Prostate testing for cancer and Treatment – trial), in which recruiters were specifically encouraged to elicit patients’ treatment preferences and trained in how to manage them, showed that it was possible to understand initially-expressed preferences and establish that many were not well-founded [14, 15]. After sensitive exploration of patients’ treatment preferences many preferences dissipated sufficiently so that many were open to randomization [15], leading to higher levels of recruitment with high rates of allocation acceptance [14, 19]. The details of how this was achieved in the ProtecT trial compared with how preferences are managed in other trials form the focus of the present research. The aims were to: (1) compare how treatment preferences were elicited and managed in recruitment consultations in three contrasting RCTs - two where trial recruiters did not receive specific training in exploring them and one (the ProtecT trial) in which recruiters were trained in how to manage them; and (2) to illustrate how strategies used by the trained recruiters in the ProtecT trial might be used to inform methods to optimize trial recruitment.

## Methods

### Randomized controlled trials

Trial 1 was undertaken in 11 UK-based hospitals to compare the effectiveness of three surgical treatments (traditional surgery versus two minimally invasive procedures) for a vascular condition. Study centers could opt for a two-arm design (traditional surgery versus one minimally invasive procedure) if preferred. Patients aged 18 years or older with the condition were referred by their GP to a routine outpatient clinic consultation with a surgeon for examination and a decision as to whether surgical treatment was appropriate. In the context of this routine outpatient appointment, eligible patients were informed about the different treatments available and the RCT. They were provided with patient information sheets about the trial and treatments to take home (where possible a summary information sheet was provided with the clinic invite letter in advance of the main information sheet). Around a week later a research nurse would follow-up with a telephone call to ascertain their willingness to participate in the trial. No specific training was given in relation to treatment preferences beyond the standard training for the trial. A qualitative study of recruitment was initiated in one centre that had opted for the two-arm trial design and recruitment appointments over an eight-month-period were audio-recorded to understand reasons for low levels of recruitment. Feedback from this was not given to the trial team until after the eight-month-period of audio-recording.

Trial 2 was conducted across three centers in the UK to determine the feasibility of an RCT comparing current standard non-surgical and surgical treatments for a type of cancer. Eligible patients aged 18 years or older with a recent diagnosis were invited to a clinic for a consultation with a surgeon where the two standard treatments were discussed, the study introduced, and a patient information sheet explaining the trial and treatments given. A week later a second appointment was conducted with an oncologist for further discussion of the two treatments and the trial, and to seek consent to participate. The recruiters did not have any specific training regarding how to deal with patients’ treatment preferences at recruitment. There was an integrated qualitative study of recruitment across the three centers, part of which involved audio-recording all recruitment consultations to monitor them and address any problems, but formal feedback and training regarding patients’ treatment preferences had not been performed at the time of the present study. In one centre some limited feedback about the content of recruitment appointments, including how to describe randomization and balancing of treatment arms, had been given.

Trial 3 (ProtecT) was undertaken in nine UK clinical centers and compared radical prostatectomy, radical conformal radiotherapy, and active monitoring in over 1,500 men aged between 50 and 69 years with localised prostate cancer [29]. Men were given a written patient information sheet at the time of prostate specific antigen (PSA) testing and diagnosis informing them of the treatments and the need for an RCT. An audio-recorded RCT information and recruitment appointment was held approximately one week after diagnosis with an experienced research nurse using a checklist to ensure essential information was covered. Nurses received ongoing training and feedback through training documents, individual and group discussions, and role play developed from findings from earlier qualitative research within the trial [14, 19] to ensure that they provided detailed and accurate information on the study and treatments and to facilitate recruitment across the centers [14, 19, 29]. As part of this training, eliciting and exploring treatment preferences was actively encouraged to assist men in reaching an informed decision about trial participation or the selection of treatment outside the trial.

### Data sampling, collection, and analysis

Audio-recordings of recruitment appointments in trials 1 and 2 were scrutinized and those in which participants expressed a treatment preference were included in the present study. In trial 3 (ProtecT), a sample of 93 recruitment appointments from all nine study centers over a three-month-period (taken in year six of the nine year recruitment period) had already been assessed in terms of the presence or absence of participant-expressed treatment preferences [15]. From this sample we selected all those in which participants voiced a treatment preference at any point in the consultation. We defined a ‘treatment preference’ as any favoring or liking, to any degree, towards a particular treatment. This may relate to aspects of the treatment itself and likely outcomes, or conversely relate to a dislike of the other treatments. This definition resulted from the detailed study of preferences across the 93 trial recruitment appointments in our previous research [15]. It did not distinguish between strengths of preference as our previous research demonstrated that preferences were based on a continuum from relatively unformed views to clear and justified requests for a treatment, either of which may develop into stronger views or dissipate during discussion [15]. To assess the variation within and across the study groups, participants’ sociodemographic data were obtained from questionnaires, case study reports, and patient medical records.

Audio-recordings of recruitment appointments were transcribed verbatim, read several times, and a combination of simple content analysis [30] and more in-depth thematic analysis [31] applied to the data. Transcripts were systematically coded, supported by the qualitative data organization package Atlas.ti (Scientific Software Development GmbH, Berlin, Germany), according to how preferences were revealed, the recruiter’s response to them, and any strategies to address them. A second experienced social scientist analyzed 10% of the data independently to compare, discuss, and refine coding to enhance its reliability. Codes were scrutinized and similar codes grouped to produce themes using methods of constant comparison [31]. Data from each trial were initially analyzed separately and then findings were compared across the trials to identify differences or similarities. Analysis was an iterative and cyclical process, each time adding to or modifying existing codes and going back to previous transcripts when new themes emerged in order to look for differences or similarities. Data saturation was considered to have been reached with the given sample as no new themes were forthcoming. NM conducted the main analysis, JD reviewed and contributed to the analytical process, and data were discussed with the study team.

Ethics approval was obtained in all three trials for the discussions to be audio-recorded and analyzed for the purposes of studying the recruitment process, and written consent was obtained from all participants (see ‘Acknowledgements’ for details).

## Results

### Participant and trial details

Basic trial details are shown in Table 1. Of the audio-recorded recruitment appointments from one study centre in trial 1, 8 out of 15 participants expressed a treatment preference, and 14 out of 19 participants from two study centers in trial 2 voiced a preference in either of the consultations with the surgeons or oncologists (Table 1). In trial 3 (ProtecT), 74 out of 93 participants from all nine study centers expressed a treatment preference in recruitment consultations (Table 1). None of the participants in trials 1 and 2 who expressed an initial preference agreed to be randomized, compared with 50 out of 74 (68%) in trial 3. In all three trials preferred treatments were spread across the different available options. Characteristics of the study sample are shown in Table 2.

**Table 1:** **Comparison of the RCTs’ characteristics and recruitment processes**

	Trial 1	Trial 2	Trial 3 (ProtecT)
Study interventions	Two or three surgical treatments for a vascular condition	Surgical and non-surgical treatment for cancer	Surgery, radiotherapy, and active monitoring for localised prostate cancer
Recruiting clinicians	Surgeons (with short follow-up call by research nurse)	1st appt with surgeons, 2nd with oncologists	Research nurses
Number of consultations audio-recorded	15^a^	38	93^b^
How many attended a consultation	15	19	93
How many expressed treatment preference (%)	8 (53%)^c^	14 (74%)	74 (80%)
Number of consultations where treatment preference expressed	8	21	74
Number of patients randomized who originally stated a treatment preference (%)	0 (0%)	0 (0%)	50 (68%)
Median length of consultations in minutes (range)	16 (12-48)	32 (surgeon), 32 (oncologist), 64 combined (22-99)	56 (21-120)

^a^Consultations with the surgeons and the follow-up phone call were combined where applicable.

^b^Sample of all recruitment appointments over a three-month-period.

^c^Unable to assess 7 participants due to incomplete recordings of consultations or follow-up call.

**Table 2:** **Study sample characteristics (n = 96)**

Participant characteristics	Trial 1 (n = 8)	Trial 2 (n = 14)	Trial 3 (ProtecT) (n = 74)
	n (%)	n (%)	n (%)
Male gender	5 (63%)	8 (60%)	74 (100%)
Mean age at RCT recruitment appointment in years (SD)	51 (SD 10.8)	66 (SD 7.24)	62 (SD 4.51)
White British ethnicity	7 (88%)^a^	-^b^	72 (97%)
Married/living as married	-^b^	9 (64%)^a^	61 (82%)

^a^One observation missing.

^b^Data not available.

Participants had one or two RCT recruitment consultations, depending on the design of the trial. In trial 1, the eight participants who expressed a treatment preference had a consultation conducted by one of three surgeons, and six of these had a follow-up telephone call from the research nurse to determine their decision to participate (in the other two cases a decision not to participate was agreed at the end of the consultation with the surgeon). Appointments lasted a median of 16 minutes ranging from 12 to 48 minutes (follow-up telephone calls were combined with the recruitment consultations) (Table 1). In trial 2, the 14 participants had two recruitment consultations. The first was conducted by one of seven surgeons across the two centers and the second by one of four different oncologists. Two patients had a further appointment with the same oncologist (these consultations were combined for the analysis). A total of 28 consultations with the 14 participants were assessed and a treatment preference was expressed in 21 of these consultations. Seven of the 14 participants voiced a preference in both consultations, one did so only in the consultation with the surgeon and six did so only in the consultation with the oncologist. The median length of the recruitment appointments (combining those with the surgeon and oncologist) was 64 minutes (range 22 to 99, Table 1). Participants in trial 3 (ProtecT) had one RCT recruitment appointment ranging from 21 to 120 minutes (median 56 minutes), conducted by one or occasionally two research nurses in each study centre (Table 1).

In total, 103 recruitment appointments in which treatment preferences were expressed by 96 participants across the three RCTs were analyzed in depth to assess how recruiters responded to these preferences.

### How treatment preferences were elicited

In all three RCTs, treatment preferences tended to be expressed by participants following indirect and often open styled questions after they had been introduced to the study treatment options. Questions such as ‘what are your thoughts at the moment?’, ‘was that [patient information sheet] very clear?’, or ‘have you got any questions to start us off about the treatments?’ tended to elicit a preference. Treatment preferences were also sometimes voiced spontaneously as the recruiter began talking about a particular treatment. The RCT tended to be introduced early in trial 3 (ProtecT) consultations, as indicated in their training, but later on in the other two trials. Consequently, preferences in trial 3 consultations were often expressed comparatively earlier in the appointment.

### Recruiters’ responses to expressed treatment preferences

There was a clear difference in how recruiters responded to the expressed preference between the trials. In trial 1, participants expressed a treatment preference in either the appointments with the surgeons or the follow-up phone calls with the research nurse. Recruiters (mostly the research nurse at follow-up) readily accepted participants’ preferences without further discussion and exploration of the reason behind it in all of the eight cases:

‘RESEARCH NURSE: Have you had a chance to look through the literature you were given in the clinic about the different treatment options?

A03: Yeah I did....I mean I’d like to go for [minimally invasive surgical option].

RESEARCH NURSE: Okay that’s fine. I’ll let [recruiter’s] secretary know that.’

(Trial 1, patient chose preferred treatment)

‘A06: I’ll go for [minimally invasive surgical option]

RESEARCH NURSE: [laughs] Can I let you think about it and I’ll give you a ring. [At follow up phone call] Have you had a chance to read the literature I gave you about the study and the treatment options?

A06: I have yes

RESEARCH NURSE: Okay and what would you like to do?

A06: Um, I think I wanna go with um [minimally invasive surgical option]

RESEARCH NURSE: Okay.’

(Trial 1, patient chose preferred treatment).

Some participants, unprompted, stated the reason behind wanting a particular treatment, but in half of the cases the rationale for the preference was not apparent and it was not possible to know whether they fully understood the other treatment option or the existence of the RCT.

In trial 2, preferences were expressed then accepted by recruiters without any discussion or exploration in 8 of the 21 consultations. This occurred in both centers and by a variety of different recruiters. In one of these cases the recruiter agreed to the participant receiving their treatment of choice before providing full details on the treatment, and in another the recruiter actually agreed with the participant’s treatment choice and in doing so revealed their lack of equipoise:

‘B08: I made up my mind at the weekend when I read about the [non-surgical option]. I just don’t want it.

ONCOLOGIST 1: You don’t want it. No, no. That’s fine.’

(Trial 2, study centre 1, patient chose preferred treatment).

‘B10: Because from what I can gather it’s not that bad, it hasn’t spread anywhere else. So the chances are this, this [non-surgical option] could cure it.

ONCOLOGIST 3: That’s absolutely right

B10: You know if it had gone anywhere else I’d, I would have said straightaway well go for that [surgical option]….

ONCOLOGIST 3: Yep, yeah I understand that it’s a worrying time isn’t it. So you would rather go for the [non-surgical option]?

B10: I think that’s the best thing to do.

ONCOLOGIST 3: Yep ok, that’s absolutely fine…. I think that’s the right thing to do actually.’

(Trial 2, study centre 2, patient chose preferred treatment).

There were attempts by the recruiters to address the expressed preference in the remaining 13 recruitment consultations by balancing participants’ views on the study treatments, for example, by stating the disadvantages of their preferred treatment or the advantages of their less preferred treatment in response to a voiced preference. They also highlighted the position of clinical equipoise (not knowing what the best treatment was) and hence the rationale and importance of the trial:

‘B01: I want rid of it, to have surgery to get it - having the surgery and getting it cut away…

SURGEON 1: Well, I will tell you honestly that I do not know which is better and I’m a surgeon but I feel that - erm, that we need to try to find out which kind of treatment is better.’

(Trial 2, study centre 1, patient chose preferred treatment).

However, the discussion did not usually go beyond this initial counterbalancing and it tended not to be tailored to the individual’s specific concerns. In most cases recruiters accepted the preferred treatment soon after providing the counterbalanced information, often without discussion of the underlying rationale for the preference and therefore understanding of the reason for the preference:

‘ONCOLOGIST 3: Would you be prepared to think about letting the computer decide whether you have an op or the [non-surgical] treatment?

B05: Well we’ve had a chat with friends, family and everybody really and - I think the operation we had decided, we thought maybe it’s one operation and that’s it, it’s gone hopefully. We looked at some of the implications with the [non-surgical option], like you said they’ve all got complications with whichever one you have. But I think the operation was the favorite one

ONCOLOGIST 3: So - I - I’m, shall I just give you a contrary view? Would that be helpful?

B05: Yes, yes.

ONCOLOGIST 3: I’m quite happy if you make that decision that’s absolutely fine, ok. So there are two things one the surgery has a very high complication rate, and the quality of life probably dips more with surgery than it does with [non-surgical option], ok. It’s true that you imagine you have the cut and it’s all over and done with, but in many ways [non-surgical option] is aiming at having the same effect…… they each have their pros and their cons and they’re both very different. And that’s really why we need to do the trial so you come here and you say well look what’s the best treatment for me and I can say, well it is - because at the moment we can’t really do that you see.

[Short discussion omitted with patient’s wife about being given several treatment options]

ONCOLOGIST 3: Yep. [7 second silence] So you’re sticking with plan A then [surgery]?

B05: Yes, yeah.’

(Trial 2, study centre 2, patient chose preferred treatment).

In a minority of cases preferences were not simply accepted by the recruiters straight after providing counterbalanced information, they continued instead with further discussion of the treatments and the trial. The recruiters’ responses appeared to have a marked effect - the participants began to consider other initially less preferred treatment - but they still opted for their preferred choice in the end. In only one of the 21 consultations in which preferences were voiced did the recruiter attempt to explicitly explore the underlying rationale for the preference before then accepting it:

‘ONCOLOGIST 4: Have you had any thoughts about it [participating in the trial]?

B12: Well, I’ve had a lot of thoughts about it and it’s always been a little bit, I don’t know. It’s, I don’t know, I don’t know. But I’ve eventually, at the moment anyway, unless I heard something completely different from somebody - erm, to go for the [non-surgical option].

[Short discussion omitted on random treatment allocation]

ONCOLOGIST 4: Ok. You’d rather stick with the [non-surgical option]

B12: Go with the [non-surgical option], I think. Yes.

ONCOLOGIST 4: So, do you want to tell me why that is?

B12: Not really because, erm I don’t know enough about it to be able to, sort of, give you a - a, sort of, serious thing. I just feel that I’d rather, sort of, go for that than - than the surgery part - it’s as simple as that.

ONCOLOGIST 4: Ok. What, in particular puts you off the surgery?

B12: Erm, I’m not particularly - I don’t think I’m particularly worried about surgery, as such, erm, but I think - well, [sighs] the surgery, as I understand it, would be, sort of - cut up here, sort of, to one thing get that out and then two more places as well to move - and I think, well, is - if the [non-surgical option] can do the same thing without the cutting that’s my only reason, really [in picking it].

ONCOLOGIST 4: Ok. That’s fair enough.’

(Trial 2, study centre 2, patient chose preferred treatment).

In this trial (trial 2), preferences were more commonly expressed in the second appointment with the oncologist than in the first consultation with the surgeon, likely in part because participants had had time to absorb the information and formulate a view. However, oncologists were more likely than surgeons to readily accept a preference at face value and not explore it; oncologists accepted a preference without discussing it further in 6 out of 13 consultations compared with only 2 out of 8 consultations with surgeons in which preferences were not pursued. Consultations appeared independent of each other in terms of discussion of treatment preferences; having voiced, and in some cases discussed, a treatment preference in the first appointment with the surgeon did not appear to affect whether the oncologist in the second appointment explored the patient’s preference or accepted it at face value. As with trial 1, all of those who expressed a treatment preference in trial 2 declined trial participation in favor of their preferred treatment.

Analysis of recruitment appointments in trial 3 (ProtecT) revealed much further exploration and discussion of expressed treatment preferences overall. Participants often came to the appointment with a particular treatment in mind. The expressions of treatment preferences were not dissimilar to those in other consultations but it was the recruiters’ response to them that differed. There was only one case in which the recruiter readily accepted the participant’s preference without further discussion of it, in a similar way to those in trials 1 and 2. In this case the man offered a clear rationale for his preference and this was accepted without exploration by the recruiter. In all of the other 73 cases, however, the recruiters did not readily accept the participant’s preference but explored it further.

Although the nurse recruiters had a checklist of information to cover in the recruitment appointments, the way they actually structured the consultation in response to a preference voiced early on varied. Some recruiters acknowledged the preference and then discussed the three study treatments, exploring and addressing the preference as part of this process. These consultations tended to be more structured and led predominantly by the recruiter [17]. In other consultations, recruiters started by exploring the preference, using this to discuss the treatments in more depth, in appointments that tended to be more loosely structured and led mostly by the participant [17].

### Exploration of preferences

Three key techniques were used by recruiters in trial 3 (ProtecT) in response to participants’ treatment preferences, all of which were indicated in their training [14]. Details of these techniques are given below. The numbers indicate the order in which techniques tended to be used, although this did vary especially in the consultations that were more loosely structured. Not all approaches were used in each consultation but at least one approach was used, and often the recruiters employed multiple approaches.

1. Elicit and acknowledge the rationale for the preference

When participants voiced a preference for a particular treatment the most frequent response from recruiters was to establish the basis for it. Justifications for preferences were often given without direct prompting, but where they were not stated or were unclear, recruiters would seek to understand them with direct questioning, such as *‘*Is there any particular reason why you think that way is better than the other ways?’ or ‘Why do you think like that?’. Once a rationale was established, recruiters moved on to explore and discuss the underlying reasons and beliefs:

‘RESEARCH NURSE: I know that [surgery] is your least [preferred] treatment. Is it the fear of an operation, going through surgery, or are there things that you’d like to discuss that I might be able to perhaps relieve some anxieties about surgery?’

(Trial 3, study centre 9, patient chose initial preference).

This more detailed exploration of the basis of the preference revealed reasons that were multilayered and complex, usually internally rational and logical, and sometimes guided by lay perceptions of prostate cancer and the available treatments. For example, favoring radical therapy to ‘get [the cancer] all away’ or desiring conservative treatment because ‘if it’s not broke don’t fix it’. Some rationales were more emotive than scientifically based, for example, relating to the experiences of relatives who had died from cancer. These discussions provided the recruiter with useful information to tailor information provision to address patient concerns.

2. Balance participants’ views about treatment

After ascertaining the basis for the preference, recruiters would usually acknowledge these reasons, but then indicate that they should still go through information about all treatments to ensure that participants had the necessary information to make an informed decision:

‘RESEARCH NURSE: I appreciate what you say about monitoring and if at the end of this discussion if that’s what you feel, we will support you whatever you want to do… But before you do that I need to go through your results with you because you’ve got to be entirely clear what your results mean… there are things about the treatments, that you may not have considered…in all the positive sides about the treatments you have to know what the down sides are as well..…you have to be able to know in your own heart, that you have explored every angle....whatever decision you make you know that you will have had all the information about these treatments for you to make that decision.’

(Trial 3, study centre 9, patient randomized to initially less desired option and accepts it).

Recruiters then provided information to balance participants’ views about treatments by highlighting the disadvantages of the preferred treatment and advantages of the less desired treatments, as some recruiters did in trial 2:

‘C01: I’ve achieved my aim as in I’ll still stick to the monitoring.

RESEARCH NURSE: How will you feel about the psychological effects of monitoring? Have you thought about how that will affect you mentally? The cancer’s still there, its’ not gone away, we haven’t removed it. I need to find out that part because the, all of our men who are on monitoring it’s the biggest problem.’

(Trial 3, study centre 1, patient chose initial preference).

‘RESEARCH NURSE: Now the thing with surgery is, yes it’s a potential cure, you’re quite right about that, but the problem with it is that umm it is a major procedure.’

(Trial 3, study centre 4, patient randomized to initial preference and accepts it).

They further encouraged participants to consider a balanced view of the treatments by tailoring information to their needs, for example, providing reassurance to alleviate specific concerns and correcting inaccuracies about treatment:

‘C89: There’s no way I can do that [continue with his caring commitments] after this [prostate] operation....

RESEARCH NURSE: Right well I mean we can help with that sort of thing.... we do put things in place to help the men when they’re out of hospital. So you know, don’t discount that immediately.......’

(Trial 3, study centre 3, patient randomized to initial preference and accepts it).

‘RESEARCH NURSE: So [I’ll] talk about the radiotherapy next

C19: Oh dear, this is the worst one

RESEARCH NURSE: Is it? Why- why d’ya think it’s the worst one?

C19: I- I’ve got a brother-in-law, he jus- (−) he’s had lots of trouble with his throat [had cancer of the throat]. And he- he’s lost his teeth, hearing’s gone and, err, he’s in a hell of a state......Because it don’t just [radiate] the (−) affected zone but it sort o- ‘cause the beam seems as though it sprays a bit an’ it, err, destroys everything

RESEARCH NURSE: .....Now (−) yes, there is always that chance that that can- th- that the radiotherapy is gonna hit the surrounding tissue ...... But, these symptoms tend to be worse towards the end of the treatment, ok, and then start to improve….. We want to reduce the side effects and the risks of the surrounding soft tissue, so, by doing -, you know, having the- the hormone treatment, it helps us to- to be more precise with the radiotherapy..... Now, what you’ve got to remember is, your brother-in-law has had a very different type of cancer.... your teeth are not going to fall out with this one, nor is your hair or anything like [that] and we are looking for precision.’

(Trial 3, study centre 7, patient randomized to initially less desired option and accepts it)

They also highlighted the position of clinical equipoise to counterbalance participants’ views, emphasizing that although all treatments offer equally good survival rates but with different side-effect profiles, there was a lack of evidence to support a clear choice between them:

‘C05: That’s the one, the monitoring. Mainly because I’ve only got a small, microscopic, yeah and I think it’d be best for me, uh, to have that, to come back every two or three months. And if it does get any worse, then I would gladly have the operation.

RESEARCH NURSE: Well, unfortunately, it doesn’t quite work like that..... the thing is, we don’t know the best way to treat prostate cancer and the three treatments that we have, in terms of how long men live, are all equal. So it doesn’t matter which treatment you have, men tend to live, you know, their life expectancy out. All three of them have advantages and disadvantages to them. And if we knew what was best for you, that’s what you would get. But we don’t know that.’

(Trial 3, study centre 3, patient randomized to initially less desired option and accepts it).

3. Emphasize need for participant to consider all treatments and equipoise

Recruiters expressed empathy with the difficult situation of clinical equipoise, particularly with men who were struggling with the decision. Throughout the consultation in response to a voiced preference they emphasized the need for participants to try to keep an open mind about each of the treatments to allow them to weigh up the advantages and disadvantages and decide if they could consider being randomized or not:

‘WIFE: My son wants him to have the operation, get rid of it

C50: But as you’ve said, if it ain’t that big....

RESEARCH NURSE: We don’t know…there’s such a dilemma about the whole thing.... because they can’t answer that question which is the best .... we know that they are as good as each other the treatments and that’s the important thing. But each have advantages and disadvantages that are different from each other because they are very different treatments. And it’s just weighing up those, and then stepping back from that, so being open minded about each of the treatments is important because as I say they are very different from each other …. looking at it and saying, well will I at least consider any one of those three treatments as an option for me and then saying if that’s yes to that then let the computer pick the treatment for you, or there’s one that you really hate, let’s kind of discuss it some more or no I’d rather choose my own treatment.’

(Trial 3, study centre 1, patient randomized to initial preference and accepts it).

Recruiters checked participants’ level of equipoise with open questions such as ‘what are your feelings at the moment?’ to determine their openness to randomization. There was often evidence of the participant expressing the rationale for his preference and the recruiter offering information in response to enable them to consider a balanced view, with checking of their position in relation to equipoise and the suggestion of randomization, at various points. This form of dialogue enabled men to reconsider their original preference and learn more about the other treatments. For some, this led to a sustained and confident treatment choice, but many shifted away from their original preference and became increasingly uncertain or equivocal [15]. Some participants even chose a treatment that was different from their originally expressed preference [15]. The appointment continued until the recruiter was satisfied that the participant was sufficiently equivocal and prepared to consider all the treatments to permit randomization, or was felt to be informed enough to choose a particular treatment.

Two extracts from appointments illustrate how these key techniques were used together by recruiters in the ProtecT trial in response to a voiced treatment preference. In the first extract we see the recruiter eliciting the man’s concerns with his less preferred treatment options and then providing information to put these concerns into perspective. The recruiter further encourages a balanced view of treatments by checking he is comfortable with the potential drawbacks of his preferred treatment. Likewise, in the second example the recruiter offers balanced information in response to a preference by emphasizing the advantages of the less preferred treatment, and later by emphasizing the position of clinical equipoise and uncertain prognosis following his concerns for the less preferred treatment. In both cases the recruiters ascertain the men’s position of equipoise (twice with C85) and when it is clear what the man’s position is, a treatment is either chosen (as in the first extract) as the preference has been sustained, or randomly allocated (as in the second extract) as the preference has dissipated and the man is accepting of all treatments:

C78: I’m definitely veering towards the monitoring side of things, because why have all those additional complications, the potential for them… I’ve got a good quality of life and I would like it to continue.....

RESEARCH NURSE: So what would be your worry with surgery?

C78:…With surgery there could be complications…the catheter and impotence.

RESEARCH NURSE: …It’s difficult to say…the majority of men won’t have those problems… What’s so worrying about radiotherapy?…

C78: Well it can affect other areas like the bladder.

RESEARCH NURSE: Hmm that’s usually very short term.

C78: Is it?.....

RESEARCH NURSE: Can I just ask, how do you think you would feel if you go for the monitoring, the one you’re drawn to, if your PSA was creeping up a bit cos it’s not something that you know from one blood test we can’t suddenly decide..... and I wonder how that would be you know sort of, kind of waiting, the blood test and all that?..... [Further discussion about drawbacks of PSA monitoring]

RESEARCH NURSE: So the idea of all treatments - they’re not equal to you in anyway?

C78: No…

RESEARCH NURSE: Whatever you decide, as long as you’ve had all the information.

C78: Oh I’ve got all the information.’

(Trial 3, study centre 9, patient chose initial preference).

C85: If I went in for the operation…. then you’ve got the recovery, then you’ve got this that and the other [side effects] and then I think I’m better to leave it [have active monitoring]

RESEARCH NURSE: The guarantee with that [surgery] I would say is that they would get rid of the prostate cancer ….. you get that reassurance because you know it’s gone, the cancer’s gone [Discussion continues about all treatments and the trial]

Wife: Oh as he walked through the door he was definitely [opting for] monitoring….

RESEARCH NURSE: How do you feel [now]?

C85: I don’t know, when does the decision actually have to be made? [Discussion about the trial/randomization]

C85: Doesn’t it say in that you could be cracking a walnut with a sledgehammer and you might be-

RESEARCH NURSE: Could be but we don’t know that you see…this is the thing we might need a sledgehammer we just don’t know, that’s the problem [Continue discussion about treatments and the trial/randomization]

C85:.... I didn’t know the implications, therapies…because to be honest I just put that on the back burner…. this has been very informative….

RESEARCH NURSE: So how do you feel then, what are we going to do?

C85: I’m, I’m happy with all three so to me it would seem a crying shame not to take part in this work today…well, well they’ve all got their pluses, they’ve all got their minuses…I haven’t got a preference as such you know they’re all equal. [Randomized and told allocation] To be honest I would have been ok with any.

(Trial 3, study centre 3, patient randomized to initial preference and accepts it).

## Discussion

This study has detailed, for the first time, how recruiters deal with patients’ treatment preferences in three different RCTs, including one where recruiters were trained and supported to elicit and explore treatment preferences. The means by which preferences were elicited did not differ greatly between the three trials; there was either a simple indirect open styled question that revealed the patient’s preference, or a preference was voiced unprompted after the introduction of a treatment. There were, however, very clear differences in how recruiters responded to the expressed preferences. In the ProtecT RCT (trial 3), trained recruiters explored the preferences and enabled participants to become clearer about whether their views were robust enough to be sustained or were sufficiently weak that participation in the RCT became possible [15]. Most were open to randomization following exploration of their preferences. In the other two trials, preferences were often accepted at face value without further discussion and exploration of the underlying rationale for the treatment choice. No participants who expressed a preference in these trials were randomized. This study has illustrated how key techniques, provided as part of a training package [14], have been implemented by trial recruiters to elicit and explore patients’ treatment preferences in recruitment discussions to improve levels of informed consent and consideration of trial participation. Such techniques could be further evaluated in a comparative study and form the basis for training and empowering recruiters to explore preferences in future trials.

A recent systematic review of the recruitment activity of clinicians across a variety of RCTs highlighted the need for training, concluding that understanding and communicating RCT methods was a priority for future interventions to improve recruitment [32]. Findings from a survey and workshop of UK Clinical Research Collaboration registered clinical trial unit directors confirmed these findings, identifying methods for improving recruitment and in particular training for site staff as the highest priority for trials methodology research [33]. Although trial recruiters perceive patients’ treatment preferences as a barrier to recruitment [6–9], a recent synthesis of the perspective of 72 recruiters from six RCTs revealed clear obstacles and hidden challenges relating to their dual roles as clinicians and recruiters that led to their discomfort in approaching and discussing trials with patients [9, 34]. The study found that recruiters were more likely to accept patients’ treatment preferences at face value without further exploration rather than offer recruitment to the RCT if that preference accorded with their own views, and highlighted the need for recruiter training and support, particularly in the management of treatment preferences [9]. Findings from the current study support these conclusions and offer suggestions in how to better manage treatment preferences.

In the past, it has been assumed that preferences make recruitment difficult [35], and that ‘challenging’ preferences may be considered to be coercive [36]. This research shows that recruiters can be trained to elicit and address patients’ treatment preferences during RCT recruitment appointments, and that they do this more often than those without this specific training, and that this can lead to an increase in the numbers of patients who then consider and accept recruitment to the RCT [14, 15, 19]. The justification for exploring patients’ treatment preferences at trial recruitment should be to gauge their level of understanding of all treatment options to ensure that their decision whether to participate or not in the trial is well-informed. This is the ethical basis for preference exploration and this gauging of understanding should occur even if the patient enters the recruitment appointment in a position of equipoise, as they may have a weak or misinformed basis for their position.

The process of recruitment is a socially delicate, interactional undertaking, with subtle moments in appointments influencing participants’ understandings and willingness to participate [37]. Despite this, very little research has focused on the content of the interactions that lead to informed consent and RCT participation. A small number of studies have shown that doctors can struggle to explain RCTs clearly [38, 39], that shared decision-making is not always practiced [40], that some participants take part because of altruism [41], and some seem unaware that they are involved in research [42]. Recordings of recruitment appointments can permit an open debate about whether the exploration of patients’ treatment preferences should be considered coercive or an informative part of gaining fully informed consent, and where a line might be drawn. We did not assess the impact of preference exploration from the patients’ perspective because the focus of our study was on recruitment appointments, and so it will be important to include patients’ perspectives of recruitment and preference exploration in future research. These issues are complex and will need to balance formal ethical guidance with increasing demands by patients to be fully informed.

One of the challenges with training recruiters to communicate the concept of clinical equipoise and to spend time understanding patients’ preferences is the impact on the time taken in consultations in which consent to participate is sought. In this study, appointments conducted by research nurses in trial 3 (ProtecT), who were supported to explore patients’ preferences, were longer (56 minutes on average) than those conducted by surgeons and the research nurse in trial 1 (16 minutes), although similar to the combined appointments with oncologists and surgeons in trial 2 (64 minutes). The difference in time between trials 1 and 3 may be explained by the simpler nature of trial 1’s interventions for a non-life threatening condition, but may also be due to the extra time taken to understand patients’ treatment preferences in trial 3. Whilst longer consultations may lead to increased costs, the costs of trial extensions, closures, or trials never being undertaken because it is thought to be too difficult to recruit need to be considered and compared with the costs of training clinicians to recruit effectively, a skill which is likely to be transferable between RCTs. Consideration should also be given to who is best placed to recruit trial participants. A nested RCT in the ProtecT feasibility study showed that trained research nurses were as effective as doctors at recruiting to the trial and more cost-effective [43], but nurses can also experience difficulty with recruitment [9, 37]. It has yet to be formally assessed if doctors can be trained to explore treatment preferences as consistently as the trained research nurses in the ProtecT study.

Although the ways recruiters responded to patients' preferences were different between trial 3 (ProtecT) and the other trials, it is possible that the observed differences were related to factors other than the specific training. There were, for example, differences across the trials in terms of the seriousness of the condition, the risk profile of the treatments, and the backgrounds and numbers of recruiters. Trial 1 offered the least invasive treatments with relatively low-risk profiles for a non-life threatening condition, with one research nurse doing most of the actual recruiting. In this trial, preferences were readily accepted without exploration which may be a reflection of the relative ‘mildness’ of the condition and low-risk profile of both treatment options. However, it may also reflect the previously identified discomfort and difficulties that nurses may have with trial recruitment (and in particular treatment preferences) in relation to their perceived roles as a caring clinical nurse [9]. Trial 2, in contrast, offered recruitment by surgeons and oncologists to a trial with more complex treatments for a potentially life-threatening condition, with diverse and potentially debilitating side effects. Despite these differences with trial 1, a sizeable proportion of recruiters also showed a similar ready acceptance of preferences, and those that did not accept immediately did not explore the underlying rationale for the treatment choice, missing opportunities to promote more informed decision-making. These findings were in contrast to the research nurses who were trained in trial 3 to sensitively explore treatment preferences.

It must also be acknowledged that the RCTs compared here might not be representative of RCTs in general and that they were a ‘convenience’ sample. Trial 1 sought external help with recruitment after experiencing difficulties, and trial 2 included an integrated qualitative study of recruitment from the outset, but the data presented here were collected before any training on patients’ treatment preferences had been given in these trials. The issue of generalization or transferability of findings should be considered in light of the number of audio-recordings analyzed, particularly in the comparator trials. The number of available audio-recordings in trial 1 was relatively small. This trial also relied on data from only one study centre and with the same research nurse following up on patients to determine their willingness to be randomized. These issues suggest that caution in interpretation of findings may be warranted, however, similar findings were observed in the other comparator trial (trial 2) where a larger number of consultations were analyzed from a variety of clinicians across two study centers. As most trials do not routinely audio-record their trial recruitment consultations, access to such data is limited.

A strength of the study was the range of qualitative analyses undertaken on ProtecT trial data [9, 15, 17, 34, 37] and the availability of audio-recorded data of recruitment appointments across three different trials. These permitted the identification of key aspects that can lead to improved recruitment (more patients sufficiently informed to consider participation and higher recruitment rates). Methods for eliciting preferences, ascertaining and acknowledging their rationales, balancing participants’ views about treatment, empathy with the difficult situation of clinical equipoise, and retaining an open mind to all treatments, could be developed into training programs to be used in other trial contexts. The strategies need to be combined with simple communication techniques, such as using open questions, long pauses, and readily ceding the floor [17], along with detailed considerations of how to avoid coercion. Previous research suggests that training oncologists in effective communication styles and behaviors may positively impact upon RCT recruitment [44]. The question remains of how much training recruiters should receive to effectively explore treatment preferences to facilitate recruitment and informed consent - whether simple encouragement is sufficient or whether more detailed training, monitoring, and feedback on communication skills and implementation of key strategies is required.

Findings here support suggestions now being made in usual clinical practice to diagnose patients’ treatment preferences and engage patients in discussion to improve informed decision-making [45]. However, doctors are not routinely trained to address patients’ treatment preferences. Communication skills required by clinicians needed to explore preferences are not often the focus of consultations in everyday practice, therefore it is not surprising that clinicians are not routinely addressing preferences within the context of trial recruitment. The key techniques identified in this study to address treatment preferences could be applied outside of the trial context to usual clinical care.

## Conclusions

In conclusion, these data show that: (1) it is possible to train trial recruiters to elicit and address patients’ treatment preferences to help them to consider participation in the trial; and (2) they do this more consistently than recruiters who do not receive this type of training. Without specific training and guidance, recruiters are likely to accept initial preferences without understanding the reasoning behind them. This can result in recruiters missing opportunities to correct inaccuracies in understanding and potentially denying patients the opportunity to fully consider all treatments and trial participation. This study has described three key techniques that recruiters can use rather than simply accepting expressed preferences at face value. Eliciting and exploring treatment preferences, if done sensitively, can enable patients to consider issues inherent in the RCT more fully, including the position of clinical equipoise and aspects that they may have misunderstood, and thus enable them to more fully consider RCT participation.

## Authors’ information

Nicola Mills PhD: Research Fellow in Health Services Research; Jane M Blazeby FRCS: Professor of Surgery; Freddie C Hamdy FMedSci: Professor of Urology and Head of Department of Surgery; David E Neal FMedSci: Professor of Surgical Oncology; Bruce Campbell FRCS: Consultant Surgeon; Caroline Wilson PhD: Research Associate in Health Services Research; Sangeetha Paramasivan PhD: Research Associate in Health Services Research; Jenny L Donovan PhD: Professor of Social Medicine.

## Abbreviations

RCT: Randomized controlled trial
ProtecT: Prostate testing for cancer and Treatment
PSA: prostate specific antigen.
